# Supplementary material for: Myeloid-derived interleukin-1β drives oncogenic KRAS-NF-κΒ addiction in malignant pleural effusion
Source: Nat Commun. 2018 Feb 14;9:672. doi: 10.1038/s41467-018-03051-z (PMC5813197; doi:10.1038/s41467-018-03051-z)
Supplement: Supplementary file 1 — Supplementary Information [file 41467_2018_3051_MOESM1_ESM.pdf]

ONLINE SUPPLEMENTARY INFORMATION

Myeloid-derived IL-1β drives oncogenic *KRAS*-NF-κB addiction in malignant pleural effusion

Marazioti et al.

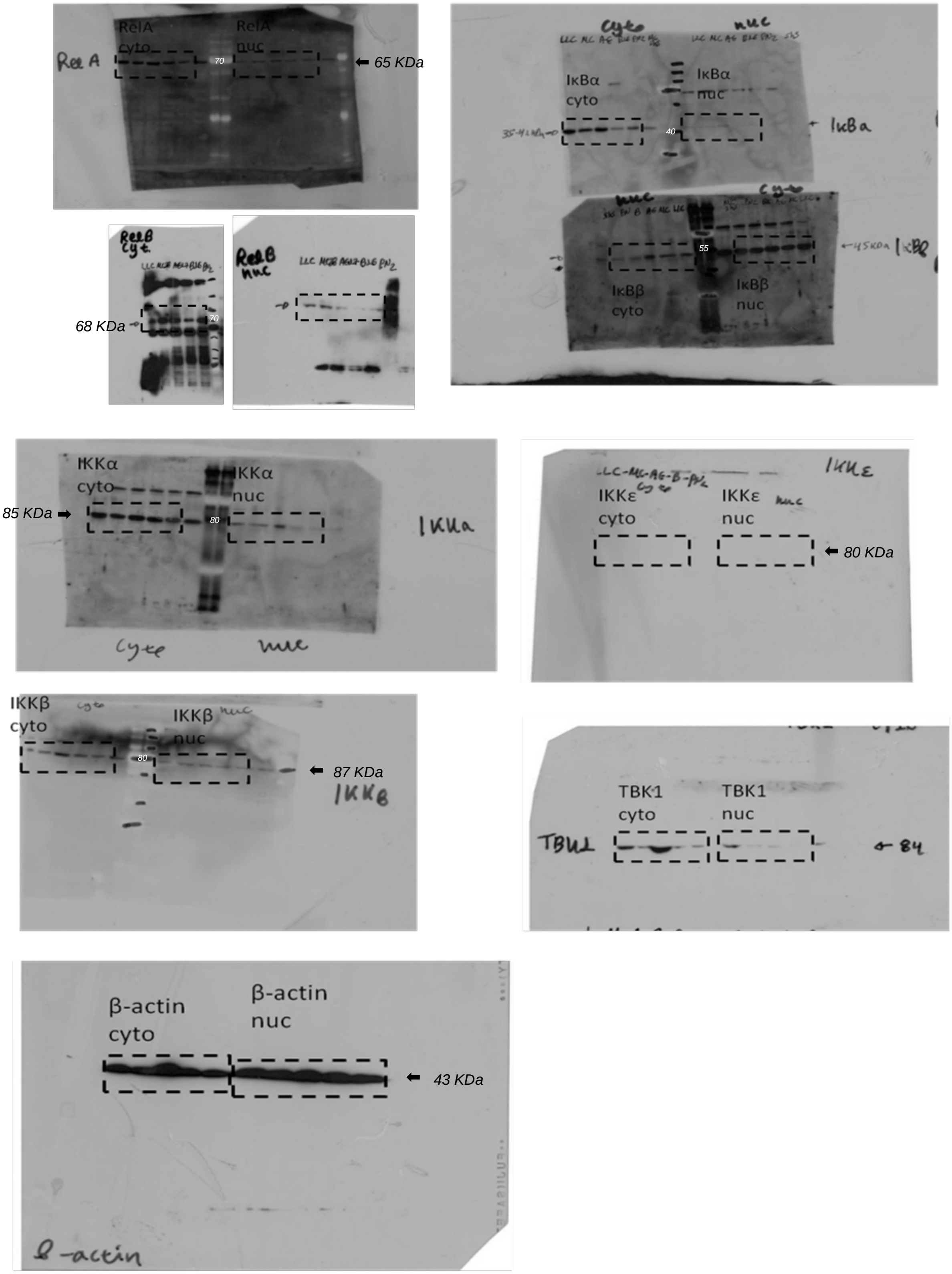

Supplementary Figure 1. Full unedited blots for **Fig. 1f**. Dashed lines indicate blot areas shown in the main Figure.

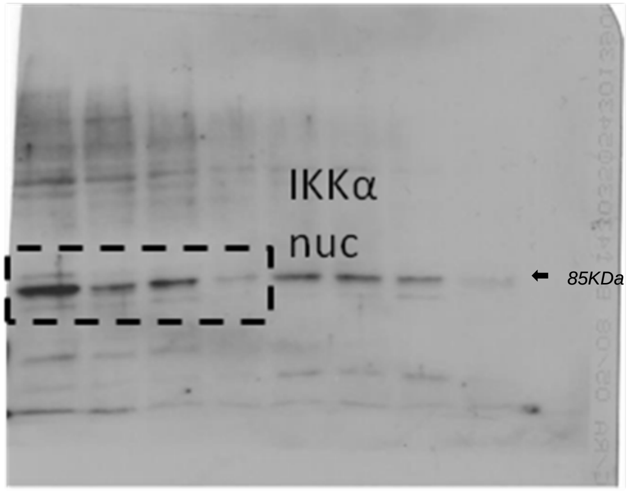

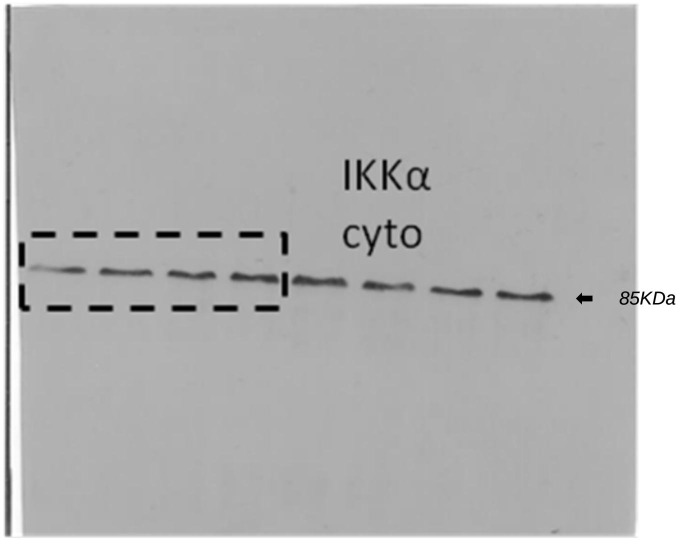

Supplementary Figure 2. Full unedited blots for **Fig. 4c**. Dashed lines indicate blot areas shown in the main Figure.

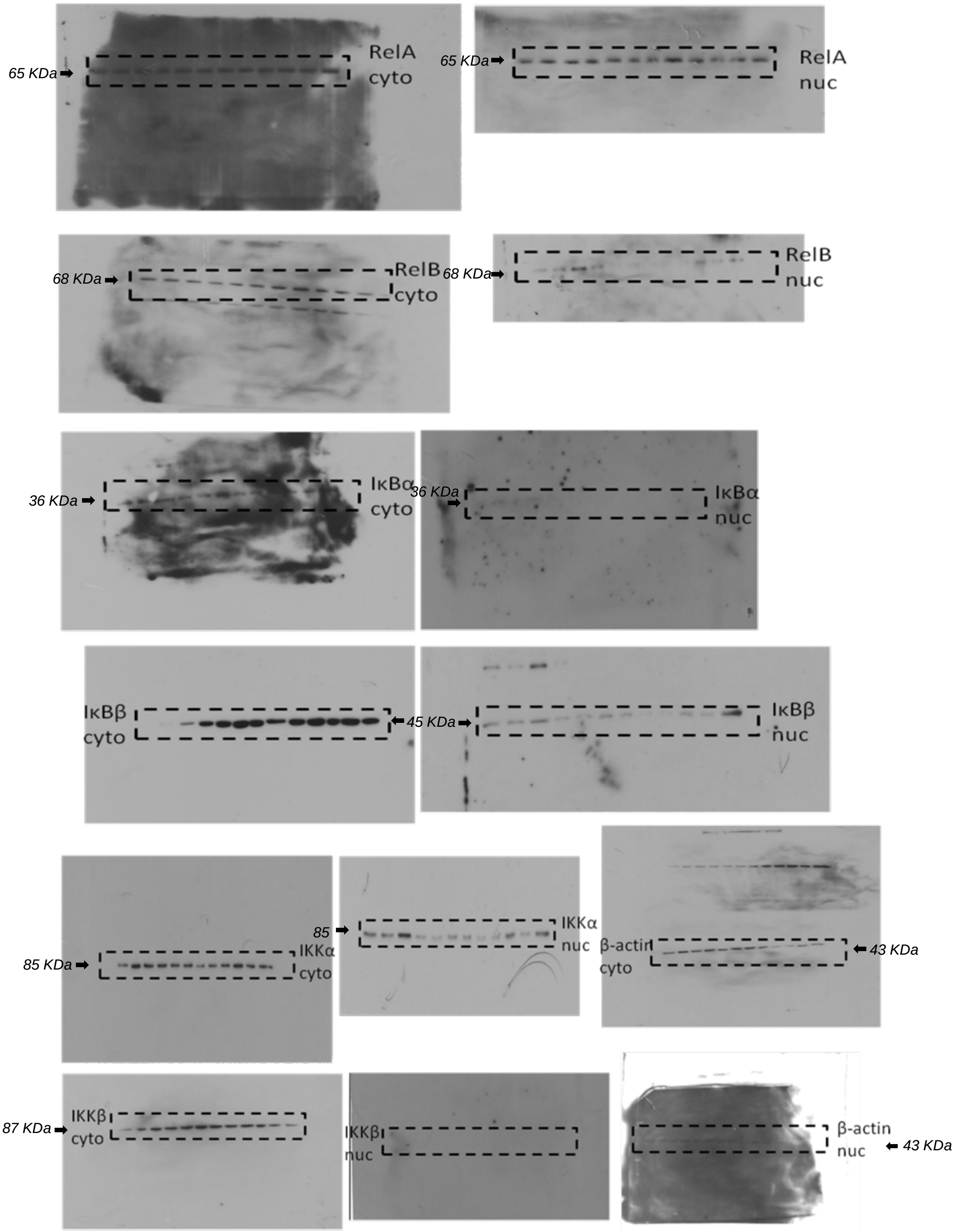

Supplementary Figure 3. Full unedited blots for **Fig. 4k**. Dashed lines indicate blot areas shown in the main Figure.

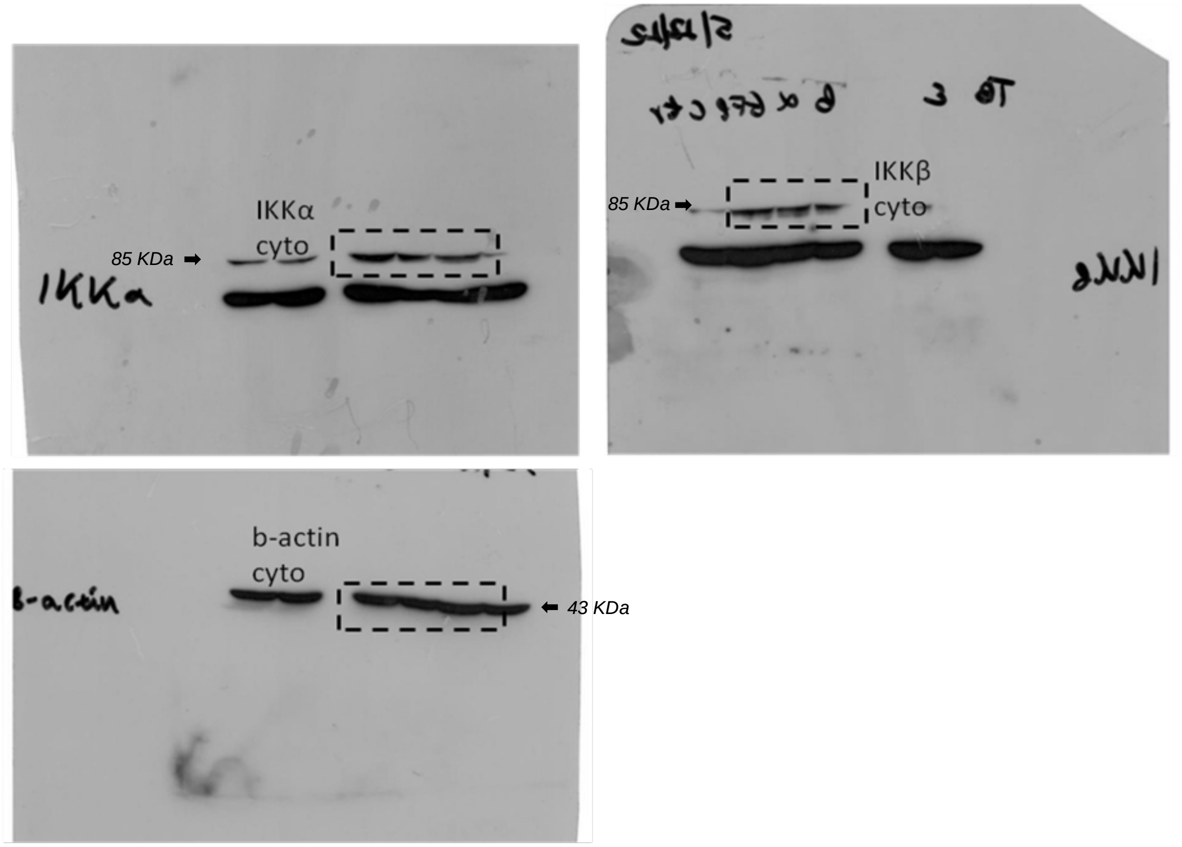

Supplementary Figure 4. Full unedited blots for **Fig. 5a**. Dashed lines indicate blot areas shown in the main Figure.

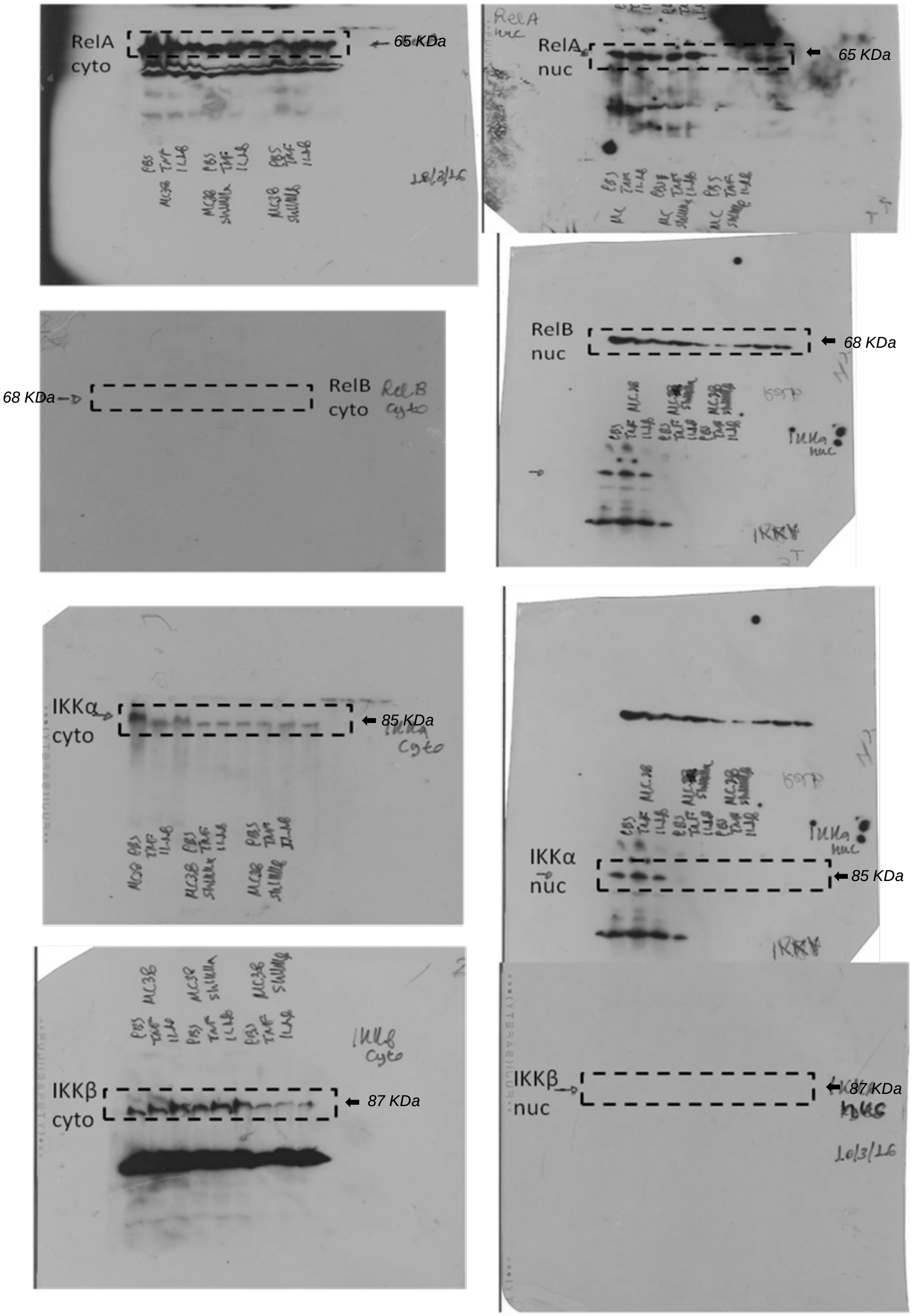

Supplementary Figure 5. Full unedited blots for **Fig. 5e**. Dashed lines indicate blot areas shown in the main Figure.

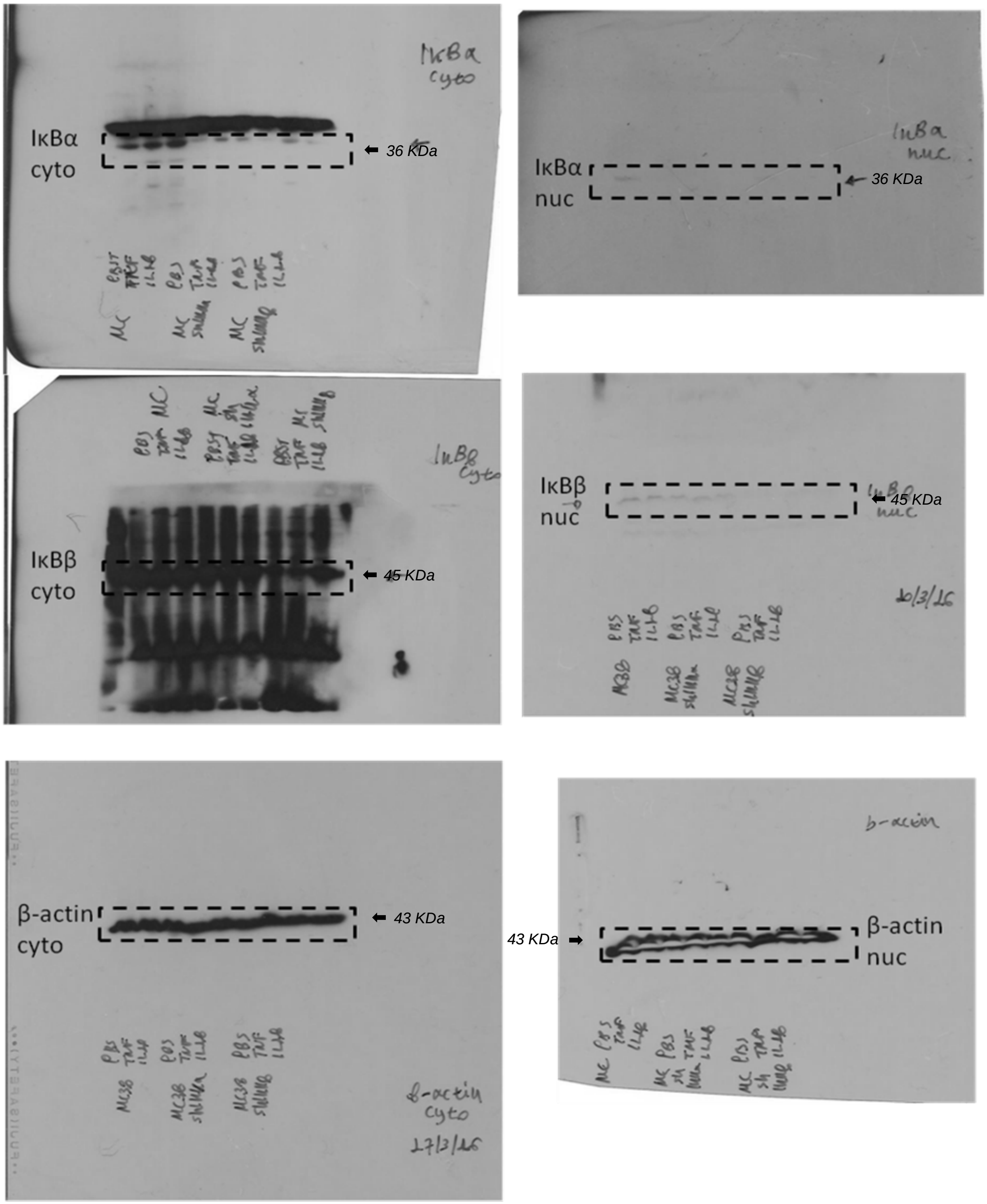

Supplementary Figure 6. Full unedited blots for **Fig. 5e** continued. Dashed lines indicate blot areas shown in the main Figure.

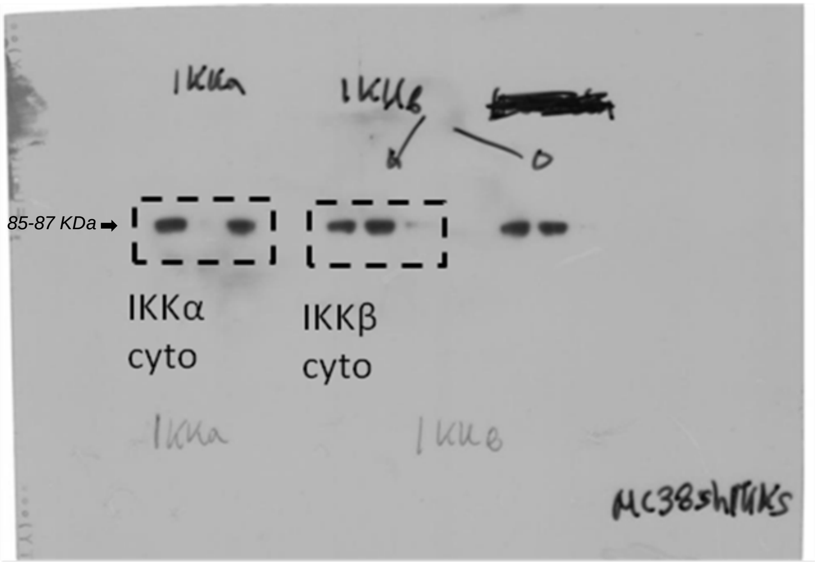

Supplementary Figure 7. Full unedited blots for **Fig. 6d**. Dashed lines indicate blot areas shown in the main Figure.

Supplementary Table 1. Inhibitory activity of the proteasome inhibitor bortezomib, the IKKβ-specific inhibitor IMD-0354, and the HSP90 and dual IKKα/IKKβ inhibitor 17-DMAG on NF-κB reporter activity and cellular proliferation of *KRAS*-mutant and *KRAS*-wild-type murine cell lines A detailed description of the experiments is given in Fig.1.

	**LLC** (*KRAS*^{G12C})	**MC38** (*KRAS*^{G13R})	**AE17** (*KRAS*^{G12C})	**B16F10** (*KRAS*^{WT})[a]	**PANO2** (*KRAS*^{WT})[a]	
	**NF-κB IC50**[b] [μM; n = 3; mean(95%CI)]					***P*** [d]
**bortezomib**	nd	nd	nd	0.12 (0.05-0.16)	0.23 (0.10-0.43)	nd
**IMD-0354**	27.55 (13.38-56.73)	9.21 (6.53-13.00)	4.04 (3.01-5.43)	0.03 (0.02-0.03)	0.03 (0.01-0.08)	< 0.0001
**17-DMAG**	0.13 (0.07-0.24)	0.57 (0.29-1.11)	0.06 (0.03-0.10)	0.04 (0.00-0.06)	0.23 (0.16-0.33)	< 0.0001
	**MTT IC50**[c] [μM; n = 3-4; mean(95%CI)]					
**bortezomib**	1.89 (1.21-2.94)	1.29 (0.86-1.94)	1.05 (0.75-1.47)	0.14 (0.09-0.20)	0.33 (0.20-0.53)	< 0.0001
**IMD-0354**	10.37 (5.66-19.00)	29.35 (11.02-78.18)	54.68 (25.08-119.20)	0.06 (0.04-0.10)	0.65 (0.33-1.26)	< 0.0001
**17-DMAG**	1.94 (0.79-4.73)	2.57 (0.86-7.73)	4.65 (2.13-10.18)	11.85 (3.50-40.18)	20.82 (8.87-48.9)	0.0042

[a] WT, wild-type.
[b] NF-κB IC_{50}, 50% inhibitory concentration of p*NGL* NF-κB reporter activity by bioluminescence imaging of live cells.
[c] MTT IC_{50}, 50% inhibitory concentration of 3-(4,5-dimethylthiazol-2-yl)-2,5-diphenyltetrazolium bromide reduction capacity.
[d] *P*, probability of no difference between cell lines by extra sum-of-squares F test.
LLC, *C57BL/6* Lewis lung carcinoma; MC38, *C57BL/6* colon adenocarcinoma; AE17, *C57BL/6* malignant pleural mesothelioma; B16F10, *C57BL/6* malignant skin melanoma; PANO2, *C57BL/6* pancreatic adenocarcinoma.

Supplementary Table 2. Inducibility of NF-κB reporter activity of *KRAS*-mutant and *KRAS*-wild-type murine cell lines by 60 different candidate ligands A detailed description of the experiments is given in Fig. 2.

**CCL11**	-	**IGF**	-	**IL-31**	-
**CCL19**	-	**IL-10**	-	**IL-33**	-
**CCL2**	-	**IL-11**	-	**IL-4**	-
**CCL20**	-	**IL-13**	-	**IL-5**	-
**CCL3**	-	**IL-15**	-	**IL-6**	-
**CCL4**	-	**IL-16**	-	**IL-7**	-
**CCL5**	-	**IL-17A**	-	**IL-9**	-
**CD135**	-	**IL-17C**	-	**LIF**	-
**CD40L**	-	**IL-17F**	-	**LPS**	LMABP
**CXCL1**	-	**IL-18**	-	**LTβ**	LMAB
**CXCL10**	-	**IL-19**	-	**MCSF**	-
**CXCL12a**	-	**IL-1α**	LMA	**NGF**	-
**CXCL12b**	-	**IL-1β**	LMA	**PDGFaa**	-
**CXCL2**	-	**IL-2**	-	**PDGFbb**	-
**EGF**	-	**IL-20**	-	**SCF**	-
**FGF1**	-	**IL-21**	-	**SPP1**	-
**FGF2**	-	**IL-22**	-	**TGF**	-
**GCSF**	-	**IL-25**	-	**TNF**	LMABP
**GMCSF**	-	**IL-27**	-	**TPO**	-
**IFN-γ**	-	**IL-3**	-	**VEGF**	-

L, induced > 2-fold in Lewis lung canrcinoma (LLC) cells (*Kras*^{G12C} mutant).
M, induced > 2-fold in MC38 colon adenocarcinoma cells (*Kras*^{G13R} mutant).
A, induced > 2-fold in AE17 malignant pleural mesothelioma cells (*Kras*^{G12C} mutant).
B, induced > 2-fold in B16F10 malignant skin melanoma cells (*Kras* wild-type).
P, induced > 2-fold in PANO2 pancreatic adenocarcinoma cells (*Kras* wild-type).

CCL; C-C motif chemokine ligand; CXCL, C-X-C motif chemokine ligand; EGF, epidermal growth factor; FGF1, fibroblast growth factor; GCSF, granulocyte colony stimulating factor; GMCSF, granulocyte-macrophage colony stimulating factor; IFN, interferon; IGF, insulin growth factor; IL, interleukin; LIF, leukemia inhibitory factor; LTβ, lymphotoxin β; MCSF, macrophage colony stimulating factor; NGF, nerve growth factor; PDGF, platelet-derived growth factor; SCF, stem cell factor; SPP, secreted phosphoprotein; TGF, transforming growth factor; TNF, tumor necrosis factor; TPO, thyroid peroxidase; VEGF, vascular endothelial growth factor.

Supplementary Table 3. Incidence of experimental malignant pleural effusions (MPE) MPE incidence in *in vivo* experiments from Figures 6-9. Shown are numbers of mice (*n*) and probability (*P*) for comparison with controls (top row of each experiment) by Fischer's exact test or for overall comparison within each experiment by χ^2 tests.

	Groups	No MPE	MPE	*P*
**Experiment from Figs. 6A-C** **LLC**-induced MPE. Cells stably expressing control or anti-IKK-specific shRNAs.	sh*C*	1	18	1.0000
	sh*Chuk*	10	6	0.0006
	sh*Ikbkb*	3	12	0.2994
	sh*Ikbke*	8	10	0.0078
	sh*Tbk1*	3	12	0.2994
				χ^2 $P = 0.0021$
**Experiment from Figs. 6D-F** **MC38**-induced MPE. Cells stably expressing control or anti-IKK-specific shRNAs.	sh*C*	1	7	1.0000
	sh*Chuk*	6	2	0.0406
	sh*Ikbkb*	2	6	1.0000
				χ^2 $P = 0.0239$
**Experiment from Figs. 6G-I** **PANO2**-induced MPE. Cells stably expressing control or IKKα, IKKβ, or mutant $Kras^{G12C}$ expression plasmids.	p*C*	9	1	1.0000
	p*Chuk*	9	1	1.0000
	p*Ikbkb*	10	0	1.0000
	p$Kras^{G1C}$	0	10	0.0001
				χ^2 $P = 0.0239$
**Experiment from Fig. 7A** **LLC**-induced MPE. Wild-type (*C57BL/6*), TNF (*Tnf-/-*), and IL-1β (*Il1b-/-*) deficient mice.	*C57BL/6*	4	14	1.0000
	Tnf-/-	1	17	0.3377
	Il1b-/-	17	1	< 0.0001
				χ^2 $P < 0.0001$
**Experiment from Fig7B** **MC38**-induced MPE. Wild-type (*C57BL/6*), TNF (*Tnf-/-*), and IL-1β (*Il1b-/-*) deficient mice.	*C57BL/6*	1	9	1.0000
	Tnf-/-	0	10	1.0000
	Il1b-/-	8	2	0.0055
				χ^2 $P = 0.0001$
**Experiment from Fig. 7E** **LLC**-induced MPE. *Il1b-/-* mice transplanted with bone marrow from wild-type, *Tnf-/-*, and *Il1b-/-* donors.	*C57BL/6*	0	10	1.0000
	Tnf-/-	2	6	0.1830
	Il1b-/-	9	2	0.0002
				χ^2 $P = 0.0004$
**Experiment from Fig. 8F** **LLC**-induced MPE. Wild-type (*C57BL/6*), CXCR1 (*Cxcr1-/-*), and CXCR2 (*Cxcr2+/-*)-deficient mice.	*C57BL/6*	0	11	1.0000
	Cxcr1-/-	5	6	0.0351
	Cxcr2+/-	5	6	0.0351
				χ^2 $P = 0.0277$
**Experiment from Fig. 9C** **LLC**-induced MPE. *C57BL/6* mice treated with daily intraperitoneal PBS, deltarasin, 17-DMAG, or deltarasin/17-DMAG (all at 15 mg/Kg in 100 μL PBS).	PBS	0	14	1.0000
	deltarasin	2	12	0.4815
	17-DMAG	2	12	0.4815
	Deltarasin + 17-DMAG	6	8	0.0159
				χ^2 $P = 0.0261$

LLC, *C57BL/6* Lewis lung carcinoma; IKK, IκB kinase; MC38, *C57BL/6* colon adenocarcinoma; Tnf, tumor necrosis factor; Il, interleukin; CXCR, C-X-C-motif chemokine receptor.

Supplementary Table 4. Microarray results from LLC cells Thirty transcripts altered more than 1.3-fold in Lewis lung adenocarcinoma cells treated with 20 ng/mL rmIL-1β (in one direction) and in Lewis lung carcinoma cells stably expressing anti-*Kras* or anti-*Chuk* shRNA (in the other direction), as assessed by microarray (mouse Gene ST2.0, Affymetrix, Sta.Clara, CA). A positive ΔGE indicates induction and a negative ΔGE suppression of a gene transcript. Gene symbols in red font were further examined in this study. A detailed description of the experiment is given in Figure 8A.

Gene symbol	Gene name	ΔGE[a] IL-1β	ΔGE[a] sh*Kras*	ΔGE[a] sh*Chuk*
Ppbp	pro-platelet basic protein	1,81	-3,00	-1,31
Cxcl1	chemokine (C-X-C motif) ligand 1	2,40	-1,93	-1,28
A4galt	alpha 1,4-galactosyltransferase	0,74	-1,08	-1,22
Wdyhv1	WDYHV motif containing 1	0,59	-0,58	-1,72
Taf1d	Tbp-associated factor, RNA polymerase I, D	0,96	-0,83	-1,08
Ifi44	interferon-induced protein 44	0,75	-0,94	-0,86
Nol10	nucleolar protein 10	1,40	-0,60	-0,41
Mmp9	matrix metallopeptidase 9	0,90	-0,45	-0,95
Caprin2	caprin family member 2	1,04	-0,58	-0,57
Dut	deoxyuridine triphosphatase	0,40	-0,54	-0,75
Fam160a1	family with sequence similarity 160, A1	-0,46	0,71	0,55
Ipo4	importin 4	-0,57	0,58	0,66
1810010H24Rik	RIKEN cDNA 1810010H24 gene	-0,61	0,83	0,49
Zfp300	zinc finger protein 300	-1,11	0,45	0,48
Camta2	calmodulin binding transcription activator 2	-0,66	0,64	0,77
Mbd6	methyl-CpG binding domain protein 6	-0,63	1,12	0,44
Spice1	spindle and centriole associated protein 1	-1,16	0,67	0,43
Scarf2	scavenger receptor class F, member 2	-1,01	0,68	0,65
Ldlr	low density lipoprotein receptor	-0,47	1,01	0,93
Rab32	RAB32, member RAS oncogene family	-0,92	0,56	1,00
Tgfb3	transforming growth factor, beta 3	-1,65	0,53	0,43
Galnt10	UDP-N-ac-α-D-galactosamine:polypeptide N-acetylgalactosaminyltransferase 10	-1,42	0,68	0,57
Neto2	neuropilin (NRP) and tolloid (TLL)-like 2	-1,06	0,44	1,74
Cpt1c	carnitine palmitoyltransferase 1c	-2,09	0,55	0,69
Arsj	arylsulfatase J	-1,82	1,00	0,87
Pmp22	peripheral myelin protein 22	-1,33	0,90	1,62
Fah	fumarylacetoacetate hydrolase	-2,04	0,91	1,12
Ephx1	epoxide hydrolase 1, microsomal	-0,96	1,62	1,52
Efnb2	ephrin B2	-2,63	1,39	0,65
Ppargc1a	peroxisome proliferative activated receptor, gamma, coactivator 1 alpha	-1,58	3,30	1,64

[a] ΔGE, difference in gene expression.

Supplementary Table 5. Microarray results from MC38 cells Twenty transcripts altered more than 1.3-fold in MC38 colon adenocarcinoma cells treated with 20 ng/mL rmIL-1β (in one direction) and in Lewis lung carcinoma cells stably expressing anti-*Kras* or anti-*Chuk* shRNA (in the other direction), as assessed by microarray (mouse Gene ST2.0, Affymetrix, Sta.Clara, CA). A positive ΔGE indicates induction and a negative ΔGE suppression of a gene transcript. Gene symbols in red font were further examined in this study. A detailed description of the experiment is given in Figure 8A.

Gene symbol	Gene name	ΔGE[a] IL-1β	ΔGE[a] sh*Kras*	ΔGE[a] sh*Chuk*
Cxcl1	chemokine (C-X-C motif) ligand 1	3,19	-0,87	-1,29
Grem1	gremlin 1	0,50	-1,79	-1,48
Mmp3	matrix metallopeptidase 3	1,92	-1,27	-0,47
Mmp13	matrix metallopeptidase 13	0,46	-0,72	-2,09
Alcam	activated leukocyte cell adhesion molecule	0,73	-1,41	-1,02
Hgf	hepatocyte growth factor	1,16	-0,86	-1,11
Creb5	cAMP responsive element binding protein 5	0,74	-1,55	-0,80
Adamts7	a disintegrin-like and metallopeptidase (reprolysin type) with thrombospondin type 1 motif, 7	0,44	-1,23	-1,01
Sema6d	sema domain, transmembrane domain (TM), and cytoplasmic domain, (semaphorin) 6D	0,45	-1,29	-0,71
Rin2	Ras and Rab interactor 2	0,41	-0,86	-1,12
Syne1	spectrin repeat containing, nuclear envelope 1	-1,31	-1,05	-2,39
Procr	protein C receptor, endothelial	0,66	-0,41	-0,86
Dgkh	diacylglycerol kinase, eta	0,56	-0,51	-0,41
LOC101056159	uncharacterized LOC101056159	-0,41	0,54	0,82
Olfr1251	olfactory receptor 1251	-0,64	0,45	0,71
H2-Q5	histocompatibility 2, Q region locus 5	-0,62	0,43	0,92
Eif4a2	eukaryotic translation initiation factor 4A2	-0,43	0,42	1,39
LOC102633783	sp110 nuclear body protein-like	-1,20	0,42	1,10
Lgals9	lectin, galactose binding, soluble 9	-1,43	1,52	1,03
Apol9b	apolipoprotein L 9b	-2,52	1,48	0,52

[a] ΔGE, difference in gene expression.

Supplementary Table 6. PCR primers used for these studies.

Method[a]	Primer	Sequence	Amplicon (bp)
qPCR	Tnfrsf1aF	CAACGTCCTGACAATGCAGA	129
qPCR	Tnfrsf1aR	CTGCATCTCCAGCCTCTCGA	
qPCR	Il1r1F	TGGAAGTCTTGTGTGCCCTT	150
qPCR	Il1r1R	GCCACATTCCTCACCAACAG	
qPCR	Il1βF	TTTGACAGTGATGAGAATGACC	162
qPCR	Il1βR	AATGAGTGATACTGCCTGCC	
qPCR	Cxcl1F	CTTGACCCTGAAGCTCCCTT	127
qPCR	Cxcl1R	GTTGTCAGAAGCCAGCGTTC	
qPCR	GusbF	TTACTTTAAGACGCTGATCACC	165
qPCR	GusbR	ACCTCCAAATGCCCATAGTC	
PCR	Mycoplasma Spp.F	GGGAGCAAACAGGATTAGATACCCT	270
PCR	Mycoplasma Spp.R	TGCACCATCTGTCACTCTGTTAACCTC	
CL	mKrasF	GGAGATCTATGACTGAGTATAAACTTGTGGTGG	583
CL	mKrasR	GGGAATTCTCACATAACTGTACACCTTGTCCTT	
CL	ChukF	ATGGGCGGCCCCCGGGGCTGCGGC	2238
CL	ChukR	TCATTCTGCTAACCAACTCCAATC	
CL	IkbkbF	ATGAGCTGGTCACCGTCCCTCCCAACCC	2274
CL	IkbkbR	TCAGTCACAGGCCTGCTCCAGGC	
CL	IkbkeF	ATGCAGAGTACCACTAACTACCTGTGGC	1900
CL	IkbkeR	TCAGACATCTGGTGCCGATGGAA	
CL	Tbk1F	ATGCAGAGCACCTCCAACCATCTGTGGC	2191
CL	Tbk1R	CTAAAGACAGTCCACATTGCGAAGGCCA	

[a]Application: PCR, DNA polymerase chain reaction; qPCR, quantitative (real-time) PCR. CL, cloning.
Provider: VBC Biotech, Vienna, Austria.

Supplementary Table 7. Antibodies used for these studies

Method[a]	Target	Provider[b]	Catalog #	Dilution	Conjugate[c]
IF,WIB	*Rel*A	Santa Cruz	Sc-372	1:200, 1:500	-
IF, WIB	*Rel*B	Santa Cruz	Sc-30887	1:200, 1:500	-
WIB	IKKα	Cell Signaling	2682	1:1000	-
WIB, ChIP	IKKβ	Cell Signaling	2684	1:1000	-
WIB	IKKε	Cell Signaling	2690	1:1000	-
WIB	TBK1	Cell Signaling	3013	1:1000	-
WIB	IκBα	Santa Cruz	Sc-371	1:500	-
WIB	IκBβ	Santa Cruz	Sc-9130	1:500	-
WIB	β-actin	Santa Cruz	sc-47778	1:500	-
WIB	Goat anti-rabbit IgG	Southern Biotech	4030-05	1:8000	HRP
WIB	Goat anti-mouse IgG	Southern Biotech	1030-05	1:8000	HRP
IF	donkey anti-rabbit & anti-mouse IgG	Invitrogen	A21206 A21202	1:1000	Alexa 488
IF	donkey anti-rabbit & anti-mouse IgG	Invitrogen	A10042 A10037	1:1000	Alexa 568
ChIP, EMSA	*Rel*A	Santa Cruz	Sc-372 X	1:60	-
ChIP, EMSA	*Rel*B	Santa Cruz	Sc-226 X	1:60	-
ChIP	IKKα	Santa Cruz	Sc-7606 X	1:60	-

[a]Application: IF, immunofluorescence; WIB, Western immunoblotting; ChIP, Chromatin Immunoprecipitation; EMSA; electrophoretic mobility shift assay
[b]Providers*:* Cell Signaling, Danvers MA, USA; Santa Cruz Biotechnology, Dallas, TX; Southern Biotech, Birmingham, AL; Invitrogen, Carlsbad, CA.
[c]Conjugates*:* HRP, horse radish peroxidase.

Supplementary Table 8. Lentiviral shRNA pools used for these studies.

**Target**	**Abbreviation**	**Catalog #**	**Target Sequences**
random	sh*C*	sc-108080-V	target sequence proprietary
Chuk	sh*Chuk*	sc-29366-V	CCATGGTGTTTGAATGTATTT<br>CTCTCAGTGTGTTCTAGATTT<br>GCAAGCAGAAGATTATTGATT
Ikbkb	sh*Ikbkb*	sc-35645-V	GATGACATCTTGAACTTGATT<br>CTGCACATTTGAATCTGTATT<br>CAGCTCTCTTAGACAGTTATT
Ikbke	sh*Ikbke*	sc-39057-V	GAGATCATGTACAGAATCATT<br>CAGTGTTGTTTGGACAAGATT<br>CCAACAAACTAGCATTACTTT
Tbk1	sh*Tbk1*	sc-39059-V	GTAGGACTGAGATATGAAATT<br>GCATCACAGAGATTTACTATT<br>GAAGTTCTAGTTTGCACAATT
Kras	sh*Kras*	sc-43876-V	CTACAGGAAACAAGTAGTA<br>GAACAGTAGACACGAAACA<br>CCATTCAGTTTCCATGTTA

Provider: Santa Cruz Biotechnology, Dallas, TX.
